# Transitions between levels of dependency among older people receiving social care – a retrospective longitudinal cohort study in a Swedish municipality

**DOI:** 10.1186/s12877-021-02283-x

**Published:** 2021-06-02

**Authors:** Magnus Zingmark, Fredrik Norström

**Affiliations:** 1 Municipality of Östersund, Health and Social Care Administration, 83182 Östersund, Sweden; 2 Department of Epidemiology and Global Health, Umeå University, Umeå, Sweden

**Keywords:** Functional decline, Disablement process, Healthy ageing, Reablement, Prevention, Health promotion

## Abstract

**Background:**

Knowledge is scarce on how needs for home help and special housing evolve among older people who begin to receive support from municipal social care. The purpose of this study was to describe baseline distributions and transitions over time between levels of dependency among older persons after being granted social care in a Swedish municipality.

**Methods:**

Based on a longitudinal cohort study in a Swedish municipality, data was collected retrospectively from municipal records. All persons 65 years or older who received their first decision on social care during 2010 (*n* = 415) were categorized as being in mild, moderate, severe, or total dependency, and were observed until the end of 2013. Baseline distributions and transitions over time were described descriptively and analysed with survival analysis, with the Kaplan-Meier estimator, over the entire follow-up period. To test potential differences in relation to gender, we used the Cox-Proportional hazards model.

**Results:**

Baseline distributions between mild, moderate, severe, and total dependency were 53, 16, 24, and 7.7%. During the first year, between 40 and 63% remained at their initial level of dependency. Among those with mild and moderate levels of dependency at baseline, a large proportion declined towards increasing levels of dependency over time; around 40% had increased their dependency level 1 year from baseline and at the end of the follow-up, 75% had increased their dependency level or died.

**Conclusions:**

Older people in Sweden being allocated home help are at high risk for decline towards higher levels of dependency, especially those at mild or moderate dependency levels at baseline. Taken together, it is important that municipalities make use of existing knowledge so that they implement cost-effective preventative interventions for older people at an early stage before a decline toward increasing levels of dependency.

## Background

Over the decades to come, larger numbers of older people in the population are expected to challenge welfare systems, since demands for health and social care are expected to increase [1]. In Sweden, the share of older people requiring social care such as home help or special housing has remained stable over time, e.g., approximately 8% of persons 65+ were allocated home help during each year from 2014 to 2019. In addition, the annual cost for such support has increased over the last 20 years, e.g., 6300 €/person 65+ and 24,500 €/person 80+ in 2019 [2]. As the number of people 80 years and older in Sweden is expected to increase by 50%, or 255,000 persons, from 2018 to 2028 [3], the effect on budgets and use of resources will be substantial. Given the association between age and increased needs, as well as costs for services to address those needs [4], it is necessary to better understand how needs emerge and develop over time in order to implement preventive measures that can improve the health and well-being of older people [5–7].

Different methods have been applied to study disability among older people and how disability changes over time. Raiche et al., followed a Canadian cohort of 1410 persons aged 75 or older at risk for decline in functioning over a four-year time-span [8]. Based on a classification system for disability that includes 14 disability profiles [9], annual probabilities of moving between these states, including recovery, stability, and decline, were calculated for the cohort [8]. In a similar longitudinal study, 1164 community-dwelling older adults (65+) were followed from baseline over 12 and 36 months to study the progression of disability in relation to three levels of functional status [10]. While these studies indicate that the proportion of older people experiencing disability increases over time, there is limited knowledge concerning how demands for home help and special housing are changing in Swedish contexts.

In Sweden, people experiencing difficulty in managing their daily activities can apply for social care, e.g., home help, from their municipality. Social care may be granted according to the Social Services Act after an application from the person is followed by a formal needs-assessment procedure led by a social worker [11]. The process of applying for and becoming in need of home help has been described as conflicting. On one hand, people can feel grateful for being provided support from the municipality, but on the other hand, they experience loss of power and independence in the process of being assessed for care needs and, subsequently, beginning to receive home help [12]. In all, the process of becoming dependent on others in activities of daily living (ADLs) has a negative impact on life satisfaction [13, 14]. This negative impact can be explained by a loss of control and restricted self-determination that limit the person’s freedom and opportunities regarding when and how to perform ADLs. Beside a loss of abilities to perform ADLs independent of others, increased dependency is also related to increased societal costs, e.g., for home help or special housing [4]. Since needs for social care, such as home help, increase in older populations, and because this has a critical impact both for individuals as well as municipalities, there is a need to better understand what kind of support first-time applicants are allocated and how their needs evolve over time.

The purpose of this study was to describe baseline distributions and transitions over time between different levels of dependency among older persons after being granted home help or special housing in a Swedish municipality.

## Methods

The study was a retrospective longitudinal cohort study conducted in a middle-sized municipality in Sweden.

### Study population

The study population included individuals 65 years or older who had their first documented contact with the municipality’s social care organization during 2010 and were granted home help or special housing. Individuals were observed until (a) the end of follow-up on December 31st 2013, (b) no more support was needed, (c) the person moved from the municipality, or (d) the person died. The data was manually extracted from the municipality’s record on decisions made by a social worker based on the Social Services Act [11]. For each decision made by a social worker, the type of support granted and the date for that decision was documented.

At the time for inclusion, there were 43 different types of support, e.g., home help or special housing that could be allocated based on a decision according to the Social Services Act in the municipality in which the study was conducted. Based on these types of support, we defined four levels of dependency (mild, moderate, severe, and total dependency) by which participants were categorized (Table 1). The categorization of dependency levels in relation to type of allocated support was done in close collaboration with a social worker experienced in working with needs assessment and social care for older people.

**Table 1:** Definitions of dependency levels in relation to the type of allocated support

Dependency level	Type of allocated support^a^
Mild	Safety alarm, food distribution, domestic tasks, e.g., cleaning, washing, making the bed
Moderate	Help with bathing, social visits, safety contact by telephone, safety visits day time or night time, escort to activities outside the home
Severe	Dressing, transferring, practical help with meals, feeding
Total	Short or long term stay at special housing
Exclusion criteria	Support to informal caregivers, daily living support^b^, day centre

^a^Examples of types of support

^b^a type of support especially targeting persons with psychiatric disability

In the mild, moderate, and severe dependency states, all participants were living in ordinary dwellings. *Mild dependency* included persons who were mainly independent but were allocated safety alarms and/or occasional help with instrumental activity of daily living (IADL). *Moderate dependency* included persons who were allocated at least one type of support included in the definition (see Table 1), but no type of support related to a higher dependency level. For severe and total dependency, participants were categorized based on the same procedure as for moderate dependency. S*evere dependency* included persons who were allocated at least one type of support with personal activities of daily living (PADL) each day. *Total dependency* included persons who were allocated support in special housing.

Three types of support in the first decision were applied as exclusion criteria: support to informal caregivers, daily living support, and day centre. Reasons for excluding these were as follows: decisions on support to informal care givers was not immediately related to the dependency level of the participants but to the overall living situation of the dyad; decision on daily living support and day centre was mainly made for persons with psychiatric or cognitive disabilities. For the second and later decisions, these decisions were considered as censoring.

For the second and later decisions, dependency levels were defined in the same way as at baseline, though *death* and *no dependency* were added as two possible states. No dependency referred to a decision where previous decisions were terminated.

### Statistical analysis

Descriptive statistics were used to present dependency levels at baseline, transitions in dependency levels between the first and second decisions, and transitions in dependency levels between the first and second year. The proportion of participants remaining at the initial level of dependency, or who had transitioned to a lower level of dependency, was analysed with survival analysis using the Kaplan-Meier estimator over the entire follow-up period, Stata version 13.1 (StataCorp LP, College Station, TX, USA). Transitions to a higher dependency level or death were considered events resulting in non-survival. Censoring was made according to the previously explained reasons. Given that gender is potentially associated with social care needs and costs [4], we explored whether there were any differences in transitions over time in relation to gender. To test potential differences in relation to gender, we used the Cox-Proportional hazards model. The proportional hazard assumption was further tested with the Schoenfeld residuals test.

## Results

**Fig. 1:**
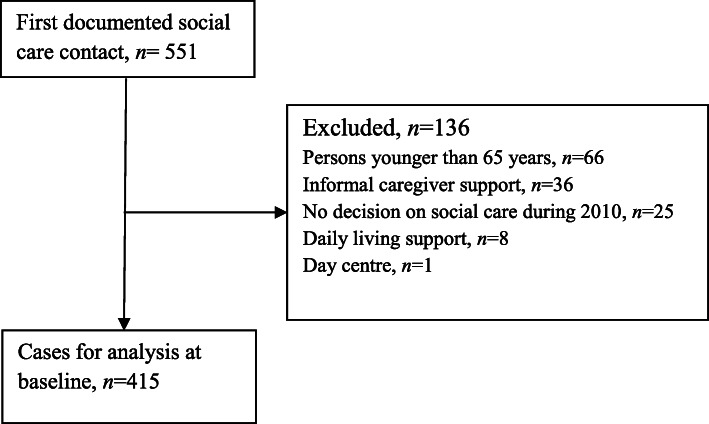
Flowchart for the inclusion of participants

During 2010, 551 individuals had their first documented contact with the municipality’s social care organization. Of these, 415 were eligible for inclusion, excluding 136 individuals who were younger than 65 years (*n* = 66), had a decision about informal caregiver support (*n* = 36), had a decision on social care that was not made during 2010 (*n* = 25), had a decision for day centre (*n* = 1), or had a decision for daily living support (*n* = 8) (See Fig. 1.)

### Dependency levels at baseline

At the time of the first decision, a majority were allocated social care equal to mild dependency (Table 2). For all levels of dependency, except for total dependency, there was a larger proportion of women.

**Table 2:** Level of dependency at first decision on social care (*n* = 415)

Dependency level^a^	***N***	%	Age (mean/median)	Women (%)
Mild	218	53	80/81	133 (61)
Moderate	66	16	80/82	40 (61)
Severe	99	24	80/82	61 (62)
Total	32	7.7	81/82	13 (41)

^a^ Dependency levels according to Table 1

### Transitions in dependency levels between first and second decision

There were 61 participants with one decision for allocated home help, of whom 34 participants were observed during the full follow-up period. Twenty-two participants decided to end their support by their own request, four participants had moved from the municipality, and one had died and, consequently, had no transition in relation to a second decision.

On average, the median time between the first and second decision was 81 days (mean 242 days) for the 354 participants with at least two decisions. In relation to baseline levels of dependency, the median time to the second decision was 221 days (mild), 59 days (moderate), 31 days (severe), and 59 days (total). For the different levels of dependency, 37–48% of the participants had the same dependency level at the second decision as in the first decision (Table 3), e.g., 48% of those initially in the severe dependency state remained there at the time of the second decision. In relation to the baseline levels of dependency, between 2 and 35% had improved to a lower level of dependency, e.g., 17% of those initially in the severe dependency state had transitioned to moderate dependency at the time of the second decision. Between 12 and 59% had a more extensive level of dependency at the time of the second decision compared to the first decision or had died by the time of the second decision, e.g., 21% of those initially in the mild dependency state had transitioned to moderate dependency at the time for the second decision. Transitions to lower levels of dependency were more prevalent among those with severe dependency at baseline, whereas transitions to higher levels of dependency were more prevalent among those with mild dependency at baseline.

**Table 3:** Dependency levels for first and second decision on social care (*n* = 354)

	Dependency level at second decision							
Dependency level at first decision	No^a^	Mild	Moderate	Severe	Total	Dead^b^	Censored^c^	In all^d^
Mild	3 (1.7%)	64 (37%)	36 (21%)	37 (22%)	13 (7.6%)	13 (7.6%)	6 (3.5%)	172
Moderate	0	8 (13%)	28 (45%)	17 (27%)	6 (9.7%)	2 (3.2%)	1 (1.6%)	62
Severe	2 (2.1%)	12 (13%)	16 (17%)	46 (48%)	8 (8.4%)	3 (3.2%)	8 (8.4%)	95
Total	0	2 (8.0%)	0	6 (24%)	11 (44%)	4 (16%)	2 (8.0%)	25
In all	5	86	80	106	38	22	17	354

^a^ No dependency according to Table 1 in the second decision of support

^b^ No second decision of support, dying during the follow-up period

^c^ Among the home care recipients, there were 17 who had support of relatives and 3 with daily living support

^d^ There were 61 home care recipients with only one decision of support at the end of the follow-up period

### Annual transitions in dependency levels

For the different levels of dependency, 40–63% of the participants had the same dependency level 1 year after the first decision as for baseline. After the first year, 34% of the participants with mild or moderate dependency at baseline had transitioned to a higher dependency level or had died (Table 4). For participants in the other baseline levels of dependency, around 20% required a higher level of support or had died 1 year after the first decision. In relation to baseline levels of dependency, between 1 and 28% had improved to a lower level of dependency after 1 year, e.g., 17% of those initially in the severe dependency state had transitioned to mild dependency 1 year after the first decision. Transitions to lower levels of dependency were more prevalent among those with severe dependency at baseline.

**Table 4:** Dependency levels 1 year after first decision on social care (*n* = 415)

	Dependency level at 1 year						
Dependency level at first decision	No^a^	Mild	Moderate	Severe	Total	Dead^b^	Censored^c^
Mild (*n* = 218)	2 (0.9%)	138 (63%)	23 (11%)	29 (13%)	11 (5.0%)	10 (4.6%)	5 (2.3%)
Moderate (*n* = 66)	1 (1.5%)	9 (14%)	33 (50%)	17 (26%)	2 (3.0%)	3 (4.5%)	1 (1.5%)
Severe (*n* = 99)	1 (1.0%)	17 (17%)	10 (10%)	40 (40%)	12 (12%)	9 (9.1%)	10 (10%)
Total (*n* = 32)	0	1 (3.1%)	1 (3.1%)	4 (13%)	18 (56%)	6 (19%)	2 (6.3%)
In all (*n* = 415)	4	165	67	90	43	28	18

^a^ No dependency according to Table 1 after 1 year

^b^ Died during the first year

^c^ Among the home care recipients, there were 18 who had support of relatives and two with daily living support

**Fig. 2:**
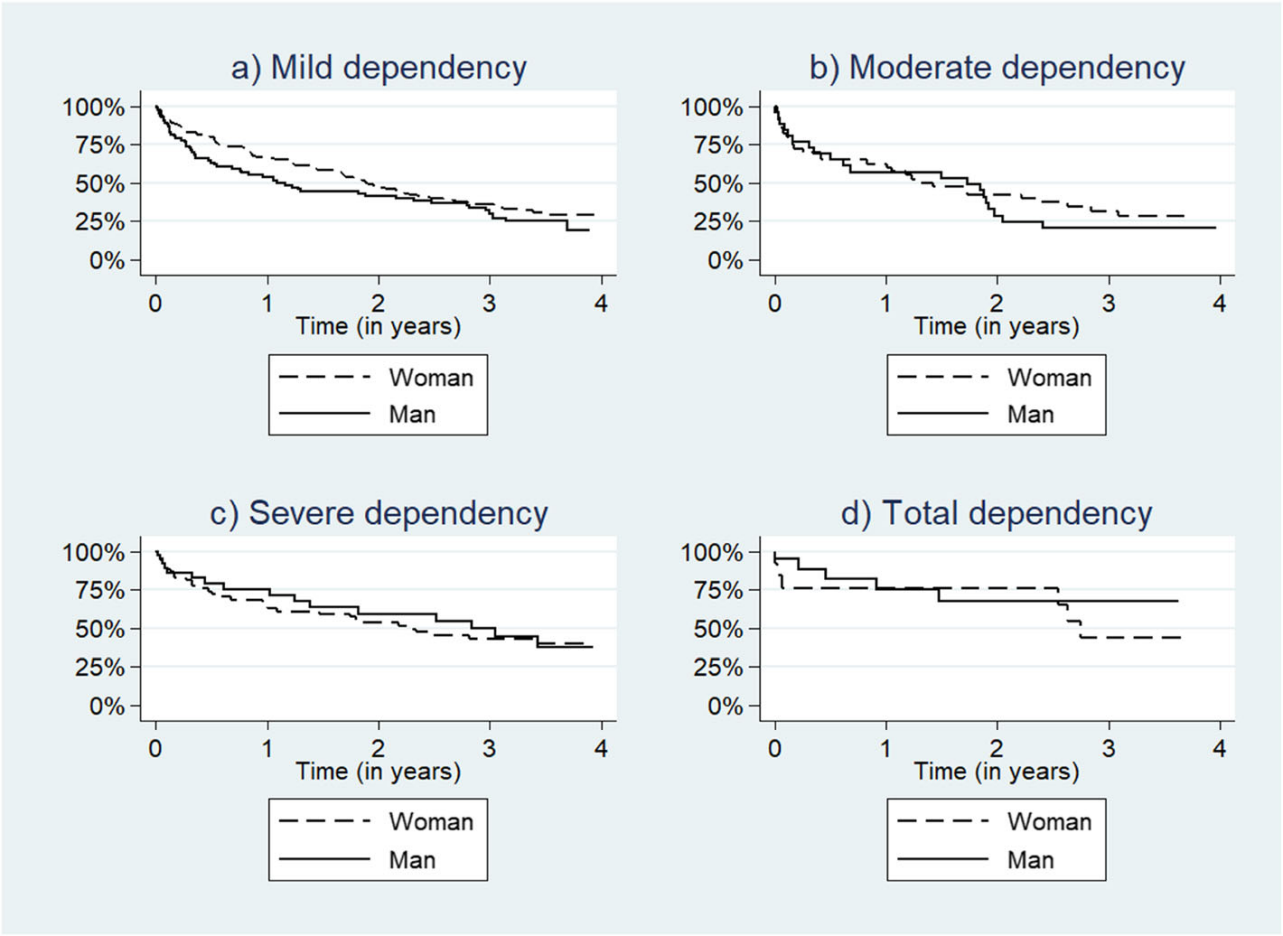
Survival function in relation to the first decision on social care for: **a** mild dependency (*n* = 133 women, 85 men), **b** moderate (*n* = 40 women, 26 men), **c** severe (*n* = 61 women, 38 men), **d** total dependency (*n* = 13 women, 19 men). Survival corresponds to the baseline dependency state or better; non-survival corresponds to a worsened state or death

In Figs. 2a-d, survival functions are presented in which survival corresponds to the proportion of participants remaining at their baseline state or a lower level of dependency. Schoenfeld’s test did not indicate a violation to the proportional hazard assumption.

For the 218 participants with mild dependency at baseline, 39% had increased their dependency level or died after 1 year (Fig. 2a). At the end of the follow-up, 75% had increased their dependency level or had died. The pattern towards higher levels of dependency was slightly different for men and women, indicating that functional decline for men was faster. A difference could not be verified when it was tested, as the difference was not statistically significant (*p* = 0.12). After 1 year, 81% of all men and 71% of all women had transitioned to a higher level of dependency or had died.

For the 66 participants with moderate dependency at baseline, 40% had increased their dependency level or had died after a year (Fig. 2b). At the end of the follow-up, 74% had increased their dependency level or had died. The pattern for transitions towards higher levels of dependency was similar during the first year for men and women but different from the second year and onwards, indicating that the functional decline for men was faster**.** However, these changes were not statistically significant (*p* = 0.67). At the end of the follow-up, 80% of all men and 72% of all women had transitioned to a higher level of dependency or had died.

For the 99 participants with severe dependency at baseline, 32% had increased their dependency level or had died after a year (Fig. 2c). At the end of the follow-up, 61% had increased their dependency level or had died. The figure indicates that the pattern towards higher levels of dependency were similar over time for men and women; however, the difference was not statistically significant (*p* = 0.31).

For the 32 participants with total dependency at baseline, 24% of the participants had died within after 1 year (Fig. 2d). At the end of the follow-up, 44% had died. The figure indicates that the pattern of transitions to death was different for men and women, However, the number of participants in total dependency was small at baseline, limiting the possibility of conducting statistical analyses.

## Discussion

Older persons who apply for and are allocated home help are at high risk for increased need of support, as indicated by the proportion transferring to higher dependency levels over time. Based on our categorization, half of all participants were at the mild dependency state at the time of the first decision. In relation to all other states, these participants were not allocated any help in relation to PADL and, in a social care context, could be considered to have minor needs. However, over time, the risk for transitions to more extensive dependency levels was high in this group. Both in relation to the first and second decision, as well as over time, the results indicate that those with relatively minor needs at the initiation of home help are part of both a large group and a group at high risk for increased dependency. A similar pattern towards increasing needs could be seen in the moderate dependency group; among participants in either mild or moderate dependency at baseline, around 75% had a higher level of dependency or had died at the end of the follow-up. Taken together, 69% of all people who begin receiving social care, i.e., those in mild or moderate dependency, are at high risk for increased needs shortly after initiation of home help.

In all groups, we could also observe transitions toward lower levels of dependency; for participants who initially were allocated home help equal to severe dependency, a larger proportion actually transferred to a lower level of dependency than those who transferred to a higher level of dependency or died during the first year. One explanation that a large proportion of participants initially in the severe dependency state recovered to milder states of dependency could be natural recovery after a hospitalization requiring extensive support during the first period after discharge. Another possible explanation could be the implementation of home health care services in the format of reablement or rehabilitation, since this type of intervention positively affects independence from home care [15, 16]. However, a lack of data on reasons for initiation of home help or parallel provision of home health care limit the possibility of drawing any firm conclusions from our data.

Our findings are to some extent in line with previous findings on how disability and dependency evolve in cohorts of older people and that both decline and improvements in functioning are frequent [8, 17]. Gill et al. followed an American cohort of initially non-disabled persons that were 70 years or older by monthly data collection over 2 years. Disability was defined in relation to the need for help in four ADLs (bathing, walking, dressing, transferring from a chair). Their results indicated that short-term disability periods followed by recovery were frequent but also that the trend over time was towards increasing disability [18]. In contrast to our results, Raîche et al. found that mild disability profiles were more stable and had less probability for deterioration than more severe profiles [8]. However, over time, deterioration towards higher levels of disability was prevalent in all profiles. Cohorts of older people have also been studied using other outcomes such as frailty (Gill 2006), and using different time periods for follow-ups, e.g., 18 months (Gill et al. 2006). While recovery towards better health/less disability occur, the overall pattern in these studies indicates that over time, the large proportion of participants decline towards poorer health/more disability [8, 17].

Our results broaden the understanding on how dependency develops over time among older people entering a Swedish social care context. When considering the increasing numbers of older people and the per-capita cost of elderly care leading to an expected economic challenge [1], our results provide a basis for discussing and planning how needs identified in social care for older people can be addressed more proactively. A critical “billion-euro question” is therefore if and how existing resources can be used more cost-efficiently to support older people. This question holds both an individual perspective in terms of promoting independence and preserving health-related quality of life [13], as well as ameliorating the expected rise on societal costs related to higher levels of disability and dependency [4].

One possible answer may be found in a recent publication by Gore et al., who introduced the framework compression of functional decline (CFD) [19]. CFD is based on an understanding of the trajectories towards functional decline and increased dependency, and how targeted interventions could be implemented at specific stages to minimize functional decline. Based on existing evidence, we know that different preventative interventions result in positive health effects for older people [20–23]. Several publications related to the “elderly in the risk zone” study in Gothenburg showed positive effects of preventive home visits and health promoting senior meetings on ADL and physical functioning for a population similar to those in the mild dependency state in our study [21, 24]. Qualitative studies provided explanations to why these interventions resulted in positive health effects, e.g., that participants described participation in health promoting senior meetings as a “key to action”, leading to initiatives towards a healthier lifestyle [25]. For persons with moderate or severe dependency, reablement and rehabilitation results in positive effects on ability to perform ADL, independence, and physical functions [26, 27]. In addition to positive health effects and reduced dependency of home care, the relatively low cost of such interventions indicate that interventions resulted in a cost-efficient use of resources [28, 29]. In a Swedish municipality context, practices such as reablement and rehabilitation are already in place, but to what extent and how well coordinated they are with social care has, to the best of our knowledge, not been explored. In contrast, preventative and health promoting interventions are not established services in Swedish municipalities. Ideally, for older people applying for social care, preventative and reablement interventions could be offered in order to preserve optimal levels of functioning and well-being. In addition, in the mild dependency group, the median time to the second decision was more than three times as long then for other levels of dependency. This may be an indicator that the time for follow-up is too extended given the high risk for decline in this group and that a shorter time to follow-up could provide better opportunities to initiate preventative measures. As proposed by Gore and colleagues, compression of functional decline could be realized if targeted interventions seeking to empower older people and optimizing functioning were implemented in time [19]. Clearly, the initiation of home help and the years thereafter seem to be critical. Future studies are needed to explore if and to what extent preventative interventions could have an impact on how older people transition towards higher levels of dependency during the years after initiation of social care.

### Methodological considerations

The data were collected retrospectively from municipal records during a period (2010–2013) in which the documentation of decisions related to social care was made in a consistent manner; from 2014 major changes in the documentation were implemented that hindered data from being collected over a more extended period of time. There was no way to extract data automatically, and therefore the data needed to be extracted manually. The task was performed by an assistant under the supervision of the first author. To ensure the quality of the extracted data, random checks were made to confirm that the correct data had been extracted. The sample was limited to one municipality, and therefore generalizability to other municipalities is very limited. In analysing transitions between levels of dependency, we acknowledge that there may be confounding factors that we did not account for, such as socioeconomic factors or diagnoses. The baseline distribution between dependency states can possibly be affected by local factors, such as access to other types of support such as health care provided by primary care or access to alternative housing options. Also, transitions between dependency states might differ due to variations in provision of preventative intervention or local routines for follow-up of decisions on social care. No data was collected concerning which extent participants received preventative interventions, rehabilitation, or reablement. However, practices such as preventive home visits or health promoting senior meetings were not part of ordinary practices at the time of data collection. In contrast, reablement was, and had been, part of the home health care provided by the municipality under study, as it is in many Swedish municipalities [30]. Three types of support were applied as exclusion criteria: support to informal caregivers, daily living support, and day centre. How care needs develop for persons receiving these types of support was beyond the scope of this study. Our data indicate that daily living support and day centre is not a common type of support being granted at the first decision. While 36 people were allocated specific support directed to informal caregivers, informal caregiving may very well have been present for other persons in the sample. While our study is based on allocated support as a proxy for the persons´ needs, we acknowledge that also other forms of support such as informal caregiving or privately financed support may have been present.

## Conclusion

Older people in Sweden being allocated social care in the format of home help are at high risk for decline towards higher levels of dependency. At the time of the first formal decision on social care, around 70% receive support equal to mild or moderate dependency. In these groups, around 40% had increased their dependency level or had died after 1 year, and by the end of the follow-up, 75% had increased their dependency level or had died. The pattern of decline in functioning is slightly different in terms of gender, indicating that men transition toward higher levels of dependency slightly faster. Taken together, it is important that municipalities make use of existing knowledge, so that they implement cost-effective preventative interventions for older people at an early stage before a decline towards increasing levels of dependency.

## Data Availability

The datasets used and/or analysed during the current study are available from the corresponding author on reasonable request.

## Abbreviations

CFD: Compression of functional decline (a framework)
IADL: Instrumental activity of daily living
PADL: Personal activity of daily living
